# Changes in Dietary Intake and Adherence to the NU-AGE Diet Following a One-Year Dietary Intervention among European Older Adults—Results of the NU-AGE Randomized Trial

**DOI:** 10.3390/nu10121905

**Published:** 2018-12-04

**Authors:** Agnes A. M. Berendsen, Ondine van de Rest, Edith J. M. Feskens, Aurelia Santoro, Rita Ostan, Barbara Pietruszka, Anna Brzozowska, Agnieszka Stelmaszczyk-Kusz, Amy Jennings, Rachel Gillings, Aedin Cassidy, Aurélie Caille, Elodie Caumon, Corinne Malpuech-Brugere, Claudio Franceschi, Lisette C. P. G. M. de Groot

**Affiliations:** 1 Division of Human Nutrition, Wageningen University & Research, Wageningen, P.O. Box 17, 6700 AA Wageningen, The Netherlands; ondine.vanderest@wur.nl (O.v.d.R.); edith.feskens@wur.nl (E.J.M.F.); lisette.degroot@wur.nl (L.C.P.G.M.d.G.); 2 Department of Experimental, Diagnostic and Specialty Medicine, Alma Mater Studiorum, University of Bologna, Via San Giacomo, 12, 40126 Bologna, Italy; aurelia.santoro@unibo.it (A.S.); claudio.franceschi@unibo.it (C.F.); 3 C.I.G. Interdepartmental Center “L. Galvani”, Alma Mater Studiorum, University of Bologna, Via G. Petroni 26, 40126 Bologna, Italy; rita.ostan3@unibo.it; 4 Department of Human Nutrition, Warsaw University of Life Sciences (WULS-SGGW), Nowoursynowska 159 C, 02-776 Warsaw, Poland; barbara_pietruszka@sggw.pl (B.P.); anna_brzozowska@sggw.pl (A.B.); adsk@o2.pl (A.S.-K.); 5 Department of Nutrition & Preventive Medicine, Norwich Medical School, University of East Anglia, Norwich Research Park, Norwich, Norfolk NR4 7TJ, UK; amy.jennings@uea.ac.uk (A.J.); R.Gillings@uea.ac.uk (R.G.); A.Cassidy@uea.ac.uk (A.C.); 6 CHU Clermont Ferrand, CRNH Auvergne, F-63000 Clermont-Ferrand, France; acaille@chu-clermontferrand.fr (A.C.); ecaumon@chu-clermontferrand.fr (E.C.); 7 Université Clermont Auvergne, INRA, UNH, Unité de Nutrition Humaine, CRNH Auvergne, F-63000 Clermont-Ferrand, France; corinne.malpuech-brugere@udamail.fr; 8 Institute of Neurological Sciences (IRCCS), Via Altura, 3, 40139 Bologna, Italy

**Keywords:** dietary intervention, Mediterranean-like diet, inflammaging

## Abstract

Background: The Mediterranean Diet has been proposed as an effective strategy to reduce inflammaging, a chronic low grade inflammatory status, and thus, to slow down the aging process. We evaluated whether a Mediterranean-like dietary pattern specifically targeting dietary recommendations of people aged over 65 years (NU-AGE diet) could be effective to shift dietary intake of older adults towards a healthful diet. Methods: Adults aged 65–80 years across five EU-centers were randomly assigned to a NU-AGE diet group or control group. The diet group followed one year of NU-AGE dietary intervention specifying consumption of 15 food groups plus the use of a vitamin D supplement. Participants in the diet group received counselling and individually tailored dietary advice, food products and a vitamin D supplement. Dietary intake was assessed by means of seven-day food records at baseline and one-year follow-up. A continuous NU-AGE index (0–160 points) was developed to assess NU-AGE diet adherence. Results: In total 1296 participants were randomized and 1141 participants completed the intervention (571 intervention, 570 control). After one year, the diet group improved mean intake of 13 out of 16 NU-AGE dietary components (*p* < 0.05), with a significant increase in total NU-AGE index (difference in mean change = 21.3 ± 15.9 points, *p* < 0.01). Conclusions: The NU-AGE dietary intervention, based on dietary recommendations for older adults, consisting of individual dietary counselling, free healthy foods and a vitamin D supplement, may be a feasible strategy to improve dietary intake in an aging European population.

## 1. Introduction

With aging of our populations [1], it is important to find modifiable strategies that slow down the aging process and its consequences in order to increase the number of years in good health in European elderly.

One of the basic molecular mechanisms of aging is the development of a chronic, low-grade inflammation status, also referred to as ‘inflammaging’ [2,3,4]. Inflammaging impacts many organs, systems and domains and is the common biological denominator of main age-related chronic diseases and geriatric syndromes, such as atherosclerosis and type 2 diabetes mellitus, and geriatric conditions, e.g., sarcopenia, frailty and disability [2,3,4].

Improving nutrition has been proposed as an effective strategy to reduce inflammaging and thus to prevent and/or postpone the onset of many diet-related diseases simultaneously. Evidence shows that, especially monounsaturated fatty acids (MUFA), polyunsaturated fatty acids (PUFA) [5,6], protein [7], vitamin D [8], vitamin B12 [9], folate [10], and anti-oxidants [11] can impact age-related health outcomes.

At the same time, it is known that with increasing age, elderly people are at higher risk of low nutrient intakes [12]. At the food group level, diets of the elderly are lacking in multiple food items, such as fruits, vegetables, and fish [13]. On the other hand, diets of the elderly are generally exceeding the requirements for saturated fatty acids [13]. Manipulating dietary patterns can be a more powerful approach than changing single nutrients or foods, because these may have synergistic or antagonistic effects when consumed in combination [14,15]. Many studies have shown that altering dietary patterns is more effective to reduce disease risk than altering single nutrients [16,17,18,19,20,21].

The Mediterranean Diet has been extensively investigated by several observational, longitudinal and randomized-controlled studies for its pivotal role in the management of a wide range of chronic age-related pathologies, such as frailty [22] and cognitive decline [23], and the reduction of all causes of mortality [24]. The Mediterranean Diet has shown antioxidant and anti-inflammatory properties largely derived from its excellent dietary fat profile characterized by a low intake of saturated fats and trans-fatty acids and an optimal ratio between omega-6 and omega-3 [25]. On the whole, the Mediterranean Diet provides an equilibrated mix of nutrients that have not only antioxidant, anti-inflammatory and prebiotic activity, but also elicits an integrated network of cellular mechanisms of stress/oxidative damage response [26].

In this context, the NU-AGE project was undertaken to investigate whether a newly designed, personally tailored Mediterranean-like dietary pattern, specifically designed to meet dietary recommendations of people over 65 years of age (NU-AGE diet), can counteract or slow down inflammaging and consequent function decline of different organs and systems in elderly [27,28].

The aim of the current study was to evaluate if a newly designed, personally tailored Mediterranean-like dietary pattern, targeting dietary recommendations of people aged over 65 years (NU-AGE diet) has been effective to shift dietary intake of apparently healthy elderly subjects from five European countries towards a healthier diet. Additionally, we evaluated if adherence to the NU-AGE guidelines is comparable among the five European sites involved within the NU-AGE study.

## 2. Materials and Methods

### 2.1. Study Design

This study was performed using baseline and follow-up data of the NU-AGE dietary intervention study [27]. The NU-AGE study is a one-year, randomized, parallel trial to investigate whether a newly designed, personally tailored Mediterranean-like dietary pattern, targeting dietary recommendations for people over 65 years of age (NU-AGE diet) can counteract or slow down the inflammaging process, as measured by C-reactive protein (CRP). A sample size of 1000 participants (80% power, two-sided alpha of 5%) was determined to detect a mean difference between groups of 0.6 in CRP (mg/L) with a standard deviation (SD) of 4 units [18]. The study was carried out in five European study centers (Bologna in Italy, Norwich in the United Kingdom (UK), Wageningen in the Netherlands, Warsaw in Poland, and Clermont-Ferrand in France). Recruitment started in April 2012 and finished in January 2014 including 1296 apparently healthy European men and women aged 65–80 years. The rationale and design of this intervention study are described in detail elsewhere [27,28]. In short, at baseline and after one-year follow-up participants completed questionnaires about their health and lifestyle and a seven-day food record to obtain information about their dietary intake. Additionally, measurements included anthropometric measurements and completion of a general questionnaire. All questionnaires and measurements were taken by trained research assistants. Local ethical approval was provided by the Independent Ethics Committee of the Sant’Orsola-Malpighi Hospital Bologna (Italy), the National Research Ethics Committee—East of England (UK), the Wageningen University Medical Ethics Committee (The Netherlands), the Bioethics Committee of the Polish National Food and Nutrition Institute (Poland) and South-East 6 Person Protection Committee (France). All study procedures were in accordance with the ethical standards of the Helsinki Declaration. All participants gave written informed consent before participating. The trial was registered at clinicaltrials.gov (NCT01754012).

### 2.2. Dietary Intervention

Participants were randomly allocated to the diet or control group to a 1:1 ratio after stratification by gender, age (65–72 or 73–79 years), frailty status (pre-frail or non-frail) and BMI (<25 or ≥25 kg/m^2^). Randomization was performed by entering the described variables of a subject into a program that automatically randomly allocates and generates a unique ID-code. 

Participants randomized into the NU-AGE diet group received monthly counselling from a trained dietician/research nutritionist and individually tailored dietary advice aiming to meet the NU-AGE Food Based Dietary Guidelines (FBDGs, Table 1). The dietary counselling covered fifteen dietary goals based NU-AGE FBDGs including a vitamin D supplement. (Table 1). To achieve changes in dietary intake by means of intrinsic motivation, dietary counselling was based on the Motivational Interview technique [29] and the Stages of Changes model [30] including five stages of change, e.g., (1) precontemplation, (2) contemplation, (3) preparation stage, (4) action and (5) maintenance. The dietician/nutritionist adapted personal advice on the participants’ stage of change, personal situations, dietary preferences, habits and beliefs.

Dietary advice was provided nine times during one year, either face-to-face or by telephone, and supported by mail or e-mail [27] using standardized procedures. During the first meeting (month 0) the dietician/research nutritionist and participant agreed on five dietary goals out of 15 NU-AGE FBDGs to work on. After one month, the participant could choose five additional dietary goals to work on until month three. At month three, the dietician/research nutritionist called the participant to evaluate the first ten goals and to implement the last set of five dietary goals. Months 5, 6 and 7 were used to optimize adherence to the NU-AGE diet. The last months of the intervention (months 9, 10 and 11) were used to motivate participant to continue the NU-AGE diet. During the whole intervention period additional information was sent out on topics that were considered important to achieve the NU-AGE FBDGs (e.g., fish consumption, food labelling, fruit and vegetable consumption, variation in foods, protein intake). Finally, at month 12 food intake and adherence was measured by means of seven-day food records. On average, the dietary counselling took six to seven hours during the one-year intervention. 

To sustain compliance to the NU-AGE FBDGs, participants received commercially available foods meeting the NU-AGE dietary guidelines. Food products included low-fat and low-salt cheese (all centers), wholegrain pasta (all centers), olive oil (all centers), and margarine rich in MUFA and PUFA (in France, UK, the Netherlands and Poland). In Italy, a frozen vegetable soup was additionally provided to participants. At the first meeting with the dietician/research nutritionist, and after 4 and 8 months, each participant in the diet group received a sufficient amount of food products for four months. In case a product could not be included in the diet, due to personal preferences, such as taste or habits, a similar product was chosen in consultation with the dietician/research nutritionist. 

Moreover, because it is not possible to achieve an adequate vitamin D intake from diet alone [31,32], participants in the NU-AGE diet group in all the study centers received a vitamin D supplement (10 µg/day). Participants in the control group received a leaflet with information on national dietary guidelines that are generally available in Italy [33], the UK [34], the Netherlands [35], Poland [36] and France [37].

**Figure 1:**
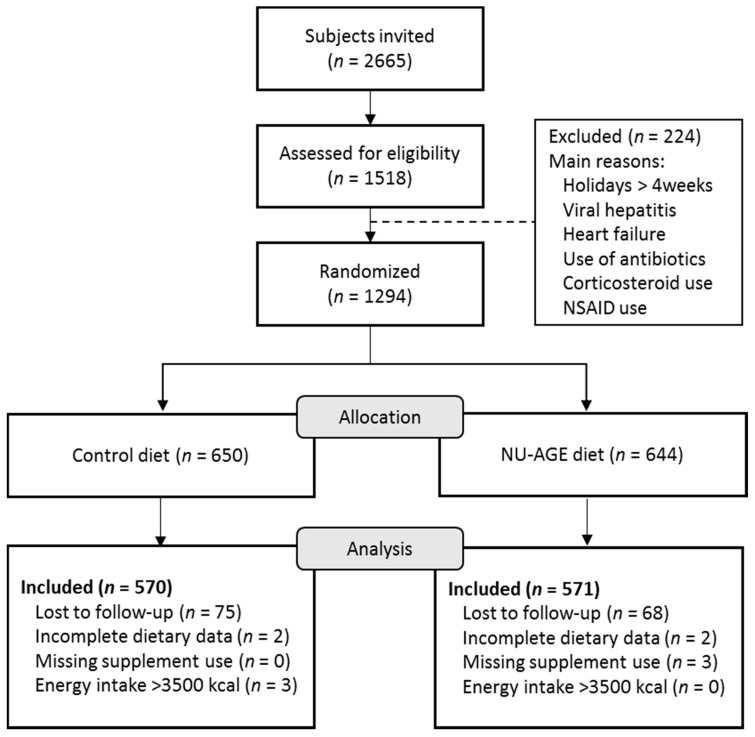
Flow chart of the recruitment process of participants in the NU-AGE dietary intervention study.

In the present study, participants who had not completed both seven-day food records at baseline and follow-up (*n* = 145), those with missing data on vitamin D supplement use (*n* = 3), incomplete dietary intake data (*n* = 4), and those with an implausible energy intake (e.g., below 500 kcal (*n* = 0), or exceeding 3500 kcal (*n* = 3) [38] were excluded. A total of 1,141 participants were included in the analysis (Figure 1; 241 participants from Italy, 252 from the UK, 241 from the Netherlands, 220 from Poland, and 187 from France).

### 2.3. Dietary Assessment

Dietary intake was estimated by means of seven-day food records completed by the participants. To remind participants to record all foods consumed, a preformatted food record was used including eight meal occasions (before breakfast, breakfast, morning snacks, lunch, afternoon snacks, evening meal, evening snacks, night snacks) referring to the current day. Participants had face-to-face training in advance to keep complete and accurate food records. Additionally, participants received written instructions about the level of detail required to describe foods and amounts consumed, including the name of the food, preparation methods, recipes for mixed foods and portion sizes [39,40]. Portion sizes were reported in national household measures, based on pictures or measured in gram or milliliters. During a one-hour interview with a trained dietician/research nutritionist the food record was reviewed and checked frequently used household measures to ensure an adequate level of detail in describing foods and food preparation methods [40]. Consumed foods were coded according to standardized coding procedures. The coding procedures comprised that all consumed foods and meals were coded into as much detail as possible. For example, a mixed dish had to be broken down into individual ingredients, including accompanying portions, and had to be coded as individual foods. Subsequently, each ingredient or food was translated into nutrients by use of local food composition tables (Nederlands voedingsstoffenbestand (NEVO) 2011 in The Netherlands [41], McCane and Widdowson’s in The UK [42], Food Composition Database for Epidemiological Studies in Italy (Banca Dati di Composizione degli Alimenti per Studi Epidemiologici in Italia, BDA) and INRAN (Istituto nazionale di ricerca per gli alimenti e la nutrizione, now called Istituto nazionale di economia agraria, CREA) in Italy [43,44], National Food and Nutrition Institute (NFNI) in Poland [45] and CIQUAL French food composition table in France [46]). Data on supplement use was obtained by means of a self-reported supplement questionnaire and checked by a trained dietician/research nutritionist.

### 2.4. Other Variables

Data on smoking status (current, former), educational level (years), employment (defined as: (1) Employed, (2) retired, or (3) other), physical activity (Physical activity scale for the elderly, PASE [47], and medical history (prevalence of hypertension [yes/no], heart disease [yes/no], diabetes mellitus type II [yes/no], hypercholesterolemia [yes/no], neurological diseases [yes/no], osteoporosis [yes/no]) were obtained by means of questionnaires. Frailty status (non-frail/pre-frail) was assessed using the frailty criteria from Fried et al. [48]. Height was measured with a stadiometer to the nearest 0.1 cm. Weight was measured to the nearest 0.1 kg with a calibrated scale while wearing light clothes. Body Mass Index (BMI) was calculated as weight/height^2^. All measures were taken by trained research assistants.

### 2.5. NU-AGE Index

#### 2.5.1. Food Based Dietary Guidelines for the Elderly Population

The NU-AGE index is meant to reflect adherence to guidance based on nutrient reference values and food based dietary guidelines for elderly individuals from Italy [33], the UK [34], the Netherlands [31,49,50,51,52], Poland [36], and France [53], on the modified MyPyramid for Older Adults [54,55], and nutrient requirements from the European Community [56], and from the Institute of Medicine [57]. These recommendations were jointly integrated into NU-AGE FBDGs in order to inform participants on a food level on what they should consume (Table 1). The NU-AGE index comprises recommendations of minimum amounts to consume for fruits, vegetables, legumes, low-fat dairy, low-fat cheese, fish, low-fat meat and poultry, nuts, olive oil, fluids, and vitamin D (from a supplement). Two recommendations give a definition of minimum and maximum intake frequencies (whole grains and low-fat meat and poultry) as extremely high intakes are not beneficial. Three recommendations refer to components to limit (alcohol, salt and sweets).

#### 2.5.2. Definition of Food Groups

Specific NU-AGE food groups were created, using country-specific food grouping systems as starting point (e.g., INRAN and BDA from Italy [43,44], WISP v4.0 from the UK [58], NEVO from the Netherlands [41], DIETA-5 from Poland [59], and CIQUAL French food composition table from France [46]. To reach a consensus on the NU-AGE food groups, expert meetings with trained dieticians/research nutritionists from all five study centers were held. All individually consumed foods were grouped into NU-AGE food groups based on the underlying principles of the NU-AGE diet (e.g., low-fat, low-sodium, high MUFA and PUFA, high fiber), resulting in harmonized food groups across countries.

#### 2.5.3. Choice of Cut-Offs and Scoring

A continuous scoring system was created to assign participants a score according to their level of adherence to each of the 16 NU-AGE diet components. This continuous scoring system allowed us to better observe changes in dietary intake compared to a dichotomous scoring system [60]. Cut-off values for each NU-AGE diet component were based on the NU-AGE FBDGs. For the adequacy components whole grains, fruits, vegetables, legumes, low-fat dairy, low-fat cheese, fish, low-fat meat and poultry, nuts, olive oil and fluids, a score ranging from 0 to 10 could be obtained for greater intakes of these components. For moderation components (alcohol, sodium and sweets), participants with lower intakes received 10 points ranging to 0 points for participants with greater intakes. The maximum level of intake for several NU-AGE diet components was established based on the country-specific population’s intake distribution (i.e., 85th percentile for sodium and sweets, 100th percentile for whole grains and low-fat meat). In total, the NU-AGE index ranged from 0 to 160 points, ranking participants according to their compliance to the NU-AGE diet. NU-AGE diet components and their cut-off values are shown in Table 1.

### 2.6. Statistical Analyses

General characteristics are reported as mean and standard deviation (SD), or number (percentage) and differences were tested using a *t*-test or chi-square test, for continuous or categorical variables, respectively. To compare changes in nutrient intake from baseline to follow-up between the NU-AGE diet group and the control group, a t-test was used, and the chi-squared test was used for comparing vitamin D supplement use. The magnitude of differences in change in dietary intake was calculated as the percentage in change from baseline intake in the control group and the NU-AGE diet group by using a *t*-test. Two categories of adherence to the NU-AGE FBDGs (Table 1) at follow-up were created; the first group did not meet the NU-AGE FBDGs and the second group had intake levels that fell within the NU-AGE FBDGs. For comparisons within countries and between countries a t-test and chi-square test were used.

All statistical analyses were carried out using SAS software version 9.4 (SAS Institute Inc., Cary, NC, USA). A two-sided *p*-value of <0.05 was considered statistically significant.

## 3. Results

### 3.1. General Characteristics

At baseline, NU-AGE participants were on average 71.0 ± 4.0 years with a BMI of 26.7 ± 3.9 kg/m^2^, and 45% of the participants were men (Table 2). Hypertension was the most frequently reported condition in both control and NU-AGE diet group (39% and 42%, respectively). In the NU-AGE diet group the proportion of pre-frail participants was significantly higher compared to the control group (24% versus 18%, *p* < 0.01).

### 3.2. Changes in Dietary Intake by Intervention Group

At baseline, the mean intake of individual components was different in the control and diet group for low-fat dairy only (155 ± 155 g and 173 ± 163 g, respectively, Table 3). The mean NU-AGE index did not differ between the control group and the NU-AGE diet group (e.g., NU-AGE index was 82.6 ± 16.5 in the control and 82.6 ± 15.3 diet group (range 36–132).

**Figure 2:**
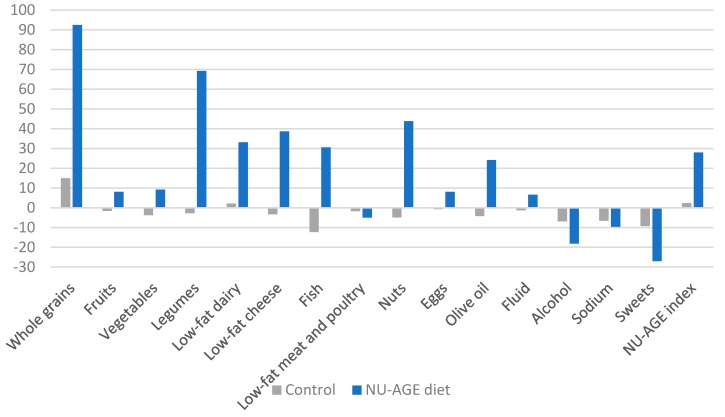
Comparison of changes in dietary intake (in % of baseline intake) after one-year NU-AGE. dietary intervention in the NU-AGE diet group and control group.

Following the one-year intervention, a significant increase in the NU-AGE index was found in the NU-AGE diet group compared to the control group (mean change 21.3 ± 15.9, *p* < 0.01, (Table 3). One-year differences in the intake of thirteen out of sixteen NU-AGE FBDGs were significantly better in the NU-AGE diet group compared to the control group (*p* < 0.05), except for low-fat meat and poultry (*p* = 0.53), eggs (*p* = 0.16) and sodium (*p* = 0.34). Major differences in changes expressed as percentages of dietary intake in the NU-AGE diet group compared to the control group were observed for whole grains (+79%), legumes (+70%), nuts (+49%), low-fat cheese (+46%), fish (+42%), low-fat dairy (+30%), and olive oil (+29%) (Figure 2).

### 3.3. Achievement of the NU-AGE Guidelines by Intervention Group

**Figure 3:**
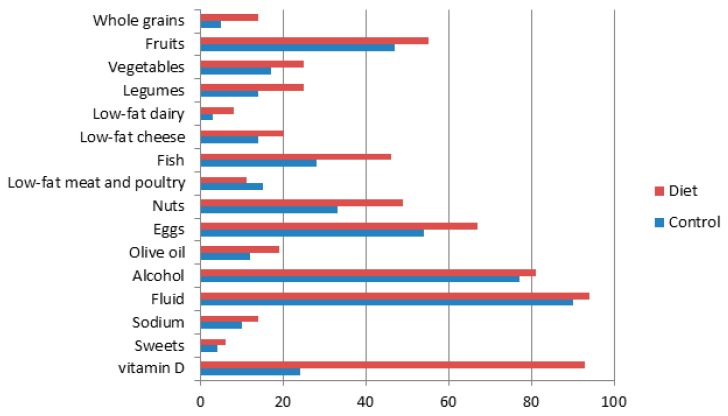
Percentage of participants in the control and NU-AGE diet group meeting the NU-AGE guidelines after one-year NU-AGE dietary intervention.

Following a one-year intervention, participants in the NU-AGE diet group achieved 6.2 ± 1.9 (range 1–12) out of 16 NU-AGE FBDGs compared to 4.5 ± 1.7 in the control group. The highest proportion meeting the NU-AGE FBDGs in both control and diet group was observed for fluid intake (90% and 94%, respectively), followed by alcohol use (77% and 81%, respectively) and egg consumption (54% and 67%, respectively) (Figure 3). The largest differences in the proportion meeting the guidelines between the NU-AGE diet and control group was observed for use of vitamin D supplement (69%), fish (18%) and nuts (16%). For low-fat meat and poultry, the proportion meeting this guideline in the NU-AGE diet group was lower at follow-up compared to the control group, possibly demonstrating an overall reduction in meat intake.

### 3.4. Country Specific Changes in Dietary Intake

At baseline, the mean NU-AGE index of participants was 84.6 ± 15.3 in Italy, 80.8 ± 14.0 in the UK, 74.5 ± 15.6 in the Netherlands, 85.4 ± 15.9 in Poland and 89.8 ± 14.4 in France. Within each country, baseline dietary intake for the control and NU-AGE diet group were similar (Table 4), whereas between countries baseline dietary intakes were different (e.g., whole grains 30.8 ± 42.9 g/day in Italy vs. 74.2 ± 61.1 g/day in Poland, vegetables 165 ± 83.9 g/day in the Netherlands vs. 282 ± 170 g/day in Poland, low-fat dairy 74.0 ± 93.1 g/day in Poland vs. 264 ± 162 g/day in the UK, olive oil 16.8 ± 8.5 g/day in Italy vs. 2.4 ± 3.5 g/day in the Netherlands). Overall, the NU-AGE index significantly improved in the NU-AGE diet group compared to the control group in all countries (*p* < 0.01). Among all study centers, French participants in the NU-AGE diet group showed the strongest improvement in adherence for 12 out of 16 NU-AGE FBGD’s, followed by the Netherlands (11 out of 16), Italy and Poland (8 out of 16), and the UK (6 out of 16).

## 4. Discussion

The NU-AGE study is a one-year randomized dietary intervention with a healthful Mediterranean-like dietary pattern targeting dietary recommendations of the aging population to reduce inflammaging, optimize health and quality of life in European older adults. In the current study we demonstrated that following the one-year NU-AGE dietary intervention, comprising individual dietary counselling, provision of free healthy foods and a vitamin D supplement, the dietary intake of the elderly European population shifts more towards the NU-AGE food based dietary guidelines (FBDGs) for a healthy diet.

Changes in dietary intake in elderly populations following an intervention have been reported in previous studies. For example, in a home-based nutrition intervention to increase consumption of fruits, vegetables, and calcium-rich foods consisting of a personalized nutrition program with home visits, phone calls and newsletters it was observed that after 6 months of intervention in community dwelling elders (aged 70 years and above), their fruit, vegetables and dairy intake improved [61]. A one-year trial including individual motivational interviews and group educational sessions by registered dieticians aiming to increase adherence to Mediterranean-type diets, demonstrated favorable changes in dietary intakes for olive oil, nuts, vegetables, legumes, fruits, meat and pastries, cakes and sweets in persons aged 55 to 80 years of age [62].

However, none of these studies investigated a dietary pattern that was specifically developed to target the dietary recommendations for the aging population. To date, dietary recommendations for the elderly population to improve healthy aging at a global level are lacking. Our study, investigating Mediterranean-like dietary pattern that was specifically designed at targeting the dietary recommendations of elderly people to fill this knowledge gap. Besides incorporating the dietary recommendations, nutrients and foods related to various aging related health outcomes and involved in chronic inflammation have been taken into account when designing the NU-AGE diet, including MUFA, PUFA, olive oil or oily fish (inflammatory processes [5] and inflammatory bowel diseases [63]), protein (sarcopenia [7]), vitamin D (falling [8]), vitamin B12 (cognitive impairment [9]), vitamin C and fruit (CRP [11]), and whole grains (type 2 diabetes [64], cardiovascular disease [65], BMI [66], and blood lipids [67]). Thus, a solid basis of evidence was used to develop the NU-AGE diet for healthier aging.

The NU-AGE dietary intervention seems to be feasible to implement in the increasingly aging population—even though this population is known to have rather stable diets over time. As demonstrated in the present study, following one year of NU-AGE intervention, participants in the NU-AGE diet group had a 21-point higher NU-AGE index (range 0–160) compared to the control group. Compared to a trial studying the Mediterranean diet in which 2 points (out of 14) change has been achieved resulting in a reduction of cardiovascular risk factors, e.g., decreased blood pressure, blood glucose levels, and cholesterol/high-density-lipoprotein (HDL) cholesterol ratio and increased HDL cholesterol levels [18], the effects of the changes in dietary intake within the NU-AGE dietary intervention seem promising to improve health at a European level.

The ability to study changes in dietary intake at a European level is a unique feature, and important strength, of the NU-AGE dietary intervention study. Even though participants in the different study centers (e.g., Italy, the UK, the Netherlands, Poland and France) had different baseline dietary intakes of the NU-AGE FBDGs, we did observe beneficial changes in dietary intake in all countries. To the best of our knowledge, no other dietary intervention study has taken this multi-country approach. 

Our approach to change the whole dietary pattern, rather than single foods or nutrients is another strength of the NU-AGE dietary intervention study. Changing an entire dietary pattern has been shown to be the preferred method as it is easier to change a dietary pattern rather than focusing on specific nutrients [68]. Moreover, we were able to achieve changes in dietary intake by making use of dietary counselling, the provision of free foods and a vitamin D supplement only. It has been demonstrated by a review that the provision of free foods is effective to improve adherence [69]. The use of Motivational Interviewing [29] in the dietary counselling could be the key to success in this intervention. A systematic review and meta-analysis has demonstrated that interventions incorporating behavior change techniques are effective ingredients of interventions to change dietary intakes [70]. The provision of free foods and a vitamin D supplement were also used to improve compliance. It has been shown that free provision of foods is effective in improving dietary habits [71,72]. Thus, with relatively less effort, without making use of a controlled feeding trial, the NU-AGE intervention seemed to be successful in making older adults change their dietary habits—even after only one year of follow-up.

Moreover, the construction of the NU-AGE diet index enables us to rank participants according to their adherence to the NU-AGE diet. This approach will allow us to study the effects of the changes in dietary intake on health outcomes using an intention to treat analysis (efficacy), but also by using a per protocol analysis (efficiency). The validity of the NU-AGE diet index needs to be studied in more diverse elderly populations including participants with various levels of education.

Furthermore, to make sure that all exposure and outcome measurements were conducted in the same standardized way across the five study centers, all questionnaires, procedures and tests were documented in standardized operating procedures (SOPs), and practiced and discussed during multiple training sessions prior to the start of the intervention period. Adherence to these SOPs was assessed and documented by means of a site visit by an independent researcher. Finally, the use of standardized and harmonized food based dietary guidelines across five study centers in Europe enabled us to provide a comparable dietary advice and to implement the same dietary principles at a large scale.

Our study has limitations. Firstly, the use of country specific food composition databases and nutrient calculation systems could have resulted in differences in intake between the countries as a result of different food composition databases rather than a difference in actual intake. We have improved the comparability in calculations by agreeing on standardized procedures for the coding of the food records and by organizing training sessions in which all research dieticians of each research center participated. Secondly, we had losses to follow-up—primarily pre-frail participants in the control group—likely because control participants did not receive food incentives and their frailty status could have limited their ability to comply with the intervention. Thirdly, to define adherence to the NU-AGE diet dietary intake data obtained by means of seven-day food records were used. Although this tool provides reliable data to evaluate short-term current dietary intake [73], in future, more objective biomarkers of dietary intake and status, such as 25-hydroxyvitamin D [74] could be incorporated in the NU-AGE index—not only to take into account the bioavailability, bioaccessibility and micronutrient status, but also to reduce misclassification bias as a result of recall bias in older adults [75]. Finally, participants were apparently healthy and well-educated [76,77], which limits the generalizability of our findings to the general elderly population.

Our results show that the NU-AGE dietary intervention, based on dietary recommendations for older adults, consisting of individual dietary counselling, provision of free healthy foods and a vitamin D supplement, is feasible to achieve favorable changes in dietary intake in an elderly population and to improve bone health [78] and cognitive health [79]. Future studies are needed to confirm the application and feasibility of the NU-AGE diet in other European populations. Evidence regarding the effects of this diet on health outcomes will contribute to the development of European-wide dietary guidelines for healthier aging across Europe.

## Figures and Tables

**Table 1:** Scoring criteria of the NU-AGE components based on NU-AGE Food Based Dietary Guidelines (FBDGs).

Component	Servings	Scoring			
		Minimum Score (0)	Lower Range ^b^ (1–10)	Maximum Score (10)	Upper Range ^c^ (10–1)
Wholemeal bread and wholegrain pasta or rice ^a^	Bread 4–6 servings/day (140–210 g/day) Pasta/rice 2 × 80 g/week (23 g/day)	Max	1–163g	163–233 g	233-max
Fruits	2 servings/day (240 g/day)	0 g	0–240 g	≥240 g	
Vegetables	300 g/day	0 g	0–300 g	≥300 g	
Legumes	200 g/week (29 g/day)	0 g	0–29 g	≥29 g	
Low-fat dairy	500 mL/day	0 g	0–500 g	≥500 g	
Low-fat cheese	30 g/day	0 g	0–30 g	≥30 g	
Fish	2 times 125 g/week (36 g/day)	0 g	0–36 g	≥36 g	
Low-fat meat and poultry ^a^	4 times 125 g/week (71 g/day)	Max	0–71 g	71–125 g	125-max
Nuts	2 times 20g/week (6 g/day)	0 g	0–6 g	>6 g	
Eggs	2–4 eggs/week (14–28 g/day)	0 g	0–14 g	>14g	
Olive oil	20 g/day	0 mL	0–20 mL	≥20 mL	
Fluid	1500 mL/day	<1000 mL	1000–1500 mL	>1500 mL	
vitamin D	Use supplement (10 µg/day)	No		Yes	
Alcohol	Max 2 servings/day for men and 1 serving/day for women	>10 g for women >20 g for men		≤10 g for women ≤20 g for men	
Salt ^a^	5 g/day (2000 mg/day sodium)	≥85th	0–1500 mg	1500–2000 mg	2000–85th
Sweets ^a^	Limited use	≥85th		0	0–85th

Fruit: Maximum 1 glass of fresh fruit juices (120 mL) can be counted as one portion of fruit. Nuts: Includes salted and unsalted nuts; ^a^ The cut-off value at which a participant would score 0 points was based on the 85th or 100th (max) percentile (pct) of the country-specific intake distribution as higher intakes are not necessarily better (100th pct wholegrains (g): 204 Italy, 212 UK, 343 The Netherlands, 381 Poland, 257 France; 100th pct meat and poultry (g): 187 Italy, 150 UK, 109 The Netherlands, 250 Poland, 193 France; 85th pct sodium (mg); 2416 Italy, 3070 UK, 2920 The Netherlands, 4686 Poland, 3673 France; 85th pct sweets (g): 95 Italy, 243 UK, 128 The Netherlands, 164 Poland, 95 France); ^b^ The range was divided into 10 and then points were given in proportion to the distance from the 0 point cut-off; ^c^ Calculation of points for dietary intake between the upper limit and the standard intake for maximum number of points: 10 − (intake − recommendation upper limit) × 10/standard upper limit.

**Table 2:** Baseline characteristics of the NU-AGE participants by intervention group (*n* = 1141).

	Control (*n* = 570)	NU-AGE Diet (*n* = 571)	*p*
Men	262 (46)	248 (43)	0.39
Age	71.1 ± 3.9	70.7 ± 4.0	0.11
Current occupation ^a^			0.23
Employed	19 (3.4)	24 (4.2)	
Retired	540 (94.7)	541 (94.7)	
Other	11 (2.1)	6 (1.1)	
Education (years)	12.6 ± 3.6	12.7 ± 3.6	0.62
BMI (kg/m^2^)	26.7 ± 3.7	26.7 ± 4.1	0.89
Physical activity (MET score)	135.1 ± 57.4	132.5 ± 54.9	0.43
Multivitamin use	67 (11.8)	86 (15.1)	0.10
Nutrient intake			
Energy, Kcal	1899 ± 448	1854 ± 432	0.08
Carbohydrates, EN%	46.9 ± 7.9	47.3 ± 7.2	0.29
Carbohydrates, g	221.9 ± 62.6	218.8 ± 60.2	0.41
Protein, EN%	16.3 ± 2.4	16.5 ± 2.8	0.14
Protein, g	76.3 ± 17.8	75.3 ± 17.7	0.36
Fat, EN%	34.3 ± 5.4	34.1 ± 5.5	0.56
Fat, g	72.6 ± 21.8	70.4 ± 20.8	0.08
Current smokers	21 (7.9)	26 (9.7)	0.46
Pre-frail	101 (17.8)	137 (24.0)	0.01
Prevalence of diseases			
Hypertension	222 (39.0)	242 (42.4)	0.25
Heart disease	97 (17.0)	94 (16.5)	0.58
Diabetes Mellitus type 2	26 (4.6)	29 (5.1)	0.69
Hypercholesterolemia	142 (24.9)	154 (27.0)	0.43
Neurological disease	12 (2.1)	17 (3.0)	0.39
Osteoporosis	59 (10.4)	70 (12.3)	0.25

Values are expressed as mean ± standard deviation (SD) or number (percentage); ^a^ Current occupation was defined as (1) employed (including part-time), (2) full-time and self-employed, (3) retired (including fully retired and semi-retired), or (4) other (including disability pension and other); Abbreviations: BMI; body mass index, MET: metabolic equivalent, EN%: energy percent.

**Table 3:** Changes in dietary intake (g) of NU-AGE diet components during one year in 1,141 participants in the NU-AGE study.

	Control Group(*n* = 570)				NU-AGE Diet Group (*n* = 571)				Difference in Change between Groups	
NU-AGE Components	Baseline	Follow-Up	Change	*p*	Baseline	Follow-Up	Change	*p*		*p*
Whole grains (g/day)	54.4 ± 53.9	62.6 ± 60.7	8.1 ± 57.4	0.02	55.7 ± 58.3	107.2 ± 66.4	51.5 ± 62.5	<0.01	43.3 ± 64.7	<0.01
Fruits (g/day)	260.0 ± 158.7	255.7 ± 154.0	−4.3 ± 156.3	0.64	248.2 ± 140.2	268.2 ± 140.0	20.1 ± 140.1	0.02	24.4 ± 127.3	<0.01
Vegetables (g/day)	221.4 ± 120.7	213.2 ± 125.7	−8.2 ± 123.2	0.26	214.5 ± 110.8	234.2 ± 103.7	19.7 ± 107.3	<0.01	27.9 ± 110.6	<0.01
Legumes (g/day)	11.1 ± 20.0	10.8 ± 19.2	−0.3 ± 19.6	0.77	10.4 ± 20.9	17.6 ± 21.9	7.2 ± 21.5	<0.01	7.5 ± 25.6	<0.01
Low-fat dairy (g/day)	155.0 ± 154.6	158.2 ± 177.5	3.2 ± 166.4	0.74	173.0 ± 163.2	230.3 ± 174.4	57.3 ± 168.9	<.001	54.1 ± 131.8	<0.01
Low-fat cheese (g/day)	11.9 ± 21.9	11.6 ± 19.6	−0.39 ± 20.8	0.75	12.7 ± 26.6	17.6 ± 24.9	4.9 ± 25.8	<0.01	5.3 ± 23.1	<0.01
Fish (g/day)	28.4 ± 29.3	24.9 ± 23.2	−3.5 ± 26.4	0.02	28.4 ± 25.3	37.1 ± 28.1	8.7 ± 26.7	<0.01	12.3 ± 32.4	<0.01
Low-fat meat and poultry (g/day)	41.2 ± 33.4	40.5 ± 31.4	−0.71 ± 32.4	0.71	40.5 ± 31.6	38.5 ± 27.9	−2.0 ± 29.8	0.26	−1.3 ± 34.7	0.53
Nuts (g/day)	6.4 ± 12.8	6.1 ± 9.9	−0.31 ± 11.4	0.65	5.7 ± 10.7	8.3 ± 9.4	2.5 ± 10.1	<0.01	2.8 ± 11.1	<0.01
Eggs (g/day)	17.5 ± 17.0	17.3 ± 17.2	−0.13 ± 17.1	0.90	17.3 ± 17.6	18.7 ± 13.9	1.4 ± 15.9	0.13	1.5 ± 18.4	0.16
Olive oil (g/day)	9.4 ± 9.9	8.9 ± 9.9	−0.39 ± 9.9	0.50	9.1 ± 9.2	11.3 ± 8.9	2.2 ± 9.1	<0.01	2.6 ± 8.0	<0.01
Fluid (mL/day)	2311 ± 654	2282 ± 651	−28.8 ± 652	0.46	2329 ± 671	2482 ± 657	154 ± 664	<0.01	182 ± 469	<0.01
Vitamin D, *n* (%)	81 (14.2)	138 (24.2)	75 (10)	<0.01	84 (14.7)	529 (92.6)	445 (77.9)	<0.01	370 (67.9)	<0.01
Alcohol (g/day)	9.6 ± 10.9	8.9 ± 10.9	−0.66 ± 10.9	0.31	8.8 ± 10.3	7.2 ± 9.1	−1.6 ± 9.7	<0.01	−0.92 ± 6.6	0.02
Sodium (mg/day)	2628 ± 1365	2455 ± 965	−173 ± 1182	<0.01	2554 ± 1500	2309 ± 946	−245 ± 1254	<0.01	−72.1 ± 1271	0.34
Sweets (g/day)	84.1 ± 113.7	76.3 ± 102.3	−7.8 ± 108.1	0.22	87.2 ± 103.5	63.6 ± 82.8	−23.6 ± 93.7	<0.01	15.8 ± 91.7	<0.01
NU-AGE index	82.6 ± 16.5	84.6 ± 16.1	1.9 ± 16.3	0.05	82.6 ± 15.3	105.7 ± 17.6	23.1 ± 16.2	<0.01	21.3 ± 15.9	<0.01

Values are expressed as mean values ± SD, unless otherwise indicated.

**Table 4:** Mean baseline and changes in dietary intake (g/day) of NU-AGE diet components by intervention group stratified by study center (*n* = 1141).

		Italy (*n* = 114 Control, 127 Diet)		UK (*n* = 126 Control, 126 Diet)		The Netherlands (*n* = 124 Control, 117 Diet)		Poland (*n* = 106 Control, 114 Diet)		France (*n* = 100 Control, 87 Diet)	
NU-AGE Components	Group	Baseline	Change	Baseline	Change	Baseline	Change	Baseline	Change	Baseline	Change
Whole grains (g/day)	Control	30.8 ± 42.9	0.52 ± 39.4	60.3 ± 41.1	23.5 ± 54.4 ^2^	71.4 ± 61.7	12.7 ± 60.2	74.2 ± 61.1	2.2 ± 64.4	31.8 ± 42.4	−1.7 ± 41.9
	Diet	23.1 ± 32.3	60.4 ± 41.5 ^2,b^	61.9 ± 46.4	34.2 ± 54.5 ^2^	82.5 ± 64.2	34.1 ± 67.2 ^2,b^	78.9 ± 70.0	38.3 ± 73.2 ^2,b^	28.2 ± 42.3	104 ± 54.1 ^2,b^
Fruits (g/day)	Control	293 ± 177	18.3 ± 182	259 ± 137	−2.2 ± 136	234 ± 146	6.8 ± 145	228 ± 159	−24.4 ± 152	290 ± 167	−25.4 ± 153
	Diet	278 ± 149	14.0 ± 144	259 ± 139	2.4 ± 140	235 ± 127	43.2 ± 129 ^2,a^	198 ± 121	9.5 ± 129 ^a^	273 ± 151	37.2 ± 142 ^b^
Vegetables (g/day)	Control	216 ± 113	−6.7 ± 112	208 ± 91.6	15.0 ± 97.2	165 ± 83.9	−6.9 ± 80.9	282 ± 170	−17.6 ± 183	249 ± 101	−30.9 ± 97.6 ^1^
	Diet	216 ± 101	23.2 ± 96.3 ^a^	218 ± 105	10.1 ± 106	162 ± 77.9	25.5 ± 78.1 ^2,b^	261 ± 146	11.3 ± 137	217 ± 87.6	31.6 ± 84.2 ^2,b^
Legumes (g/day)	Control	16.5 ± 23.1	−2.3 ± 21.9	17.4 ± 22.4	2.2 ± 20.9	3.8 ± 12.6	1.5 ± 12.1	4.8 ± 14.1	0.3 ± 18.0	12.9 ± 21.3	−4.2 ± 19.6
	Diet	8.7 ± 10.9	2.7 ± 13.2 ^b^	19.9 ± 25.9	3.9 ± 25.4	3.4 ± 11.9	6.5 ± 17.2 ^2,a^	10.2 ± 29.5	8.4 ± 26.1 ^1,a^	8.6 ± 15.1	17.9 ± 18.1 ^2,b^
Low-fat dairy (g/day)	Control	89.7 ± 103	1.1 ± 108	264 ± 162	31.2 ± 175	196 ± 166	8.0 ± 177	74.0 ± 93.1	−20.9 ± 110	127 ± 138	−10.0 ± 134
	Diet	87.6 ± 93.7	45.8 ± 99.9 ^2,b^	278 ± 165	31.4 ± 168	256 ± 183	40.9 ± 183 ^a^	96.5 ± 102	61.3 ± 1312 ^2,b^	134 ± 140	128 ± 156 ^2,b^
Low-fat cheese (g/day)	Control	0.17 ± 1.0	0.18 ± 2.15	2.2 ± 6.5	1.3 ± 8.2	9.6 ± 21.6	−0.6 ± 19.2	30.2 ± 32.9	−4.6 ± 30.6	21.3 ± 15.9	1.5 ± 17.3
	Diet	1.46 ± 4.87	0.93 ± 5.27	2.76 ± 6.65	1.42 ± 7.5	6.5 ± 10.6	7.7 ± 11.9 ^2,b^	38.8 ± 46.8	10.0 ± 41.6 ^a^	17.5 ± 15.4	5.0 ± 14.3 ^1^
Fish (g/day)	Control	29.9 ± 28.8	−2.2 ± 26.3	24.7 ± 23.5	5.2 ± 22.3	22.3 ± 26.2	−3.0 ± 25.5	31.4 ± 40.7	−8.9 ± 33.4	35.8 ± 23.3	−11.0 ± 22.2 ^2^
	Diet	27.1 ± 23.1	17.1 ± 27.5 ^2,b^	29.5 ± 24.3	2.3 ± 23.4 ^a^	23.9 ± 24.9	5.3 ± 22.9 ^a^	31.0 ± 31.2	12.2 ± 33.1 ^2,b^	31.0 ± 21.2	12.5 ± 21.5 ^2,b^
Low-fat meat and poultry (g/day)	Control	49.8 ± 35.9	−2.6 ± 34.8	33.7 ± 26.6	−0.83 ± 26.3	32.5 ± 25.4	−0.7 ± 24.8	33.3 ± 36.2	−0.36 ± 33.4	60.3 ± 34.1	1.3 ± 33.4
	Diet	51.4 ± 28.9	−2.1 ± 27.4	33.4 ± 31.8	−1.7 ± 28.6	31.2 ± 24.3	−1.66 ± 23.3	34.2 ± 32.3	−3.7 ± 31.6	56.1 ± 33.2	0.45 ± 29.4
Nuts (g/day)	Control	8.1 ± 14.4	−0.14 ± 12.6	6.1 ± 11.8	−0.19 ± 11.2	6.3 ± 11.6	0.2 ± 10.8	4.1 ± 8.4	0.9 ± 8.2	7.6 ± 16.7	−2.7 ± 13.8
	Diet	4.3 ± 10.7	0.00 ± 9.2	5.3 ± 8.3	4.1 ± 8.7 ^2,b^	6.8 ± 11.0	3.6 ± 11.1 ^1,a^	4.6 ± 8.2	3.3 ± 8.6 ^2,a^	8.4 ± 14.9	1.5 ± 12.3 ^a^
Eggs (g/day)	Control	7.3 ± 8.7	1.7 ± 10.2	16.6 ± 15.5	−0.64 ± 15.2	16.8 ± 17.3	0.86 ± 17.4	30.7 ± 20.3	−2.8 ± 21.0	16.9 ± 12.9	−0.1 ± 13.5
	Diet	7.8 ± 7.7	1.5 ± 7.9	17.2 ± 15.4	6.7 ± 15.1 ^2,b^	14.8 ± 15.2	2.9 ± 14.4	28.4 ± 22.9	−4.9 ± 19.2	20.2 ± 17.7	0.0 ± 15.3
Olive oil (g/day)	Control	16.8 ± 8.5	1.0 ± 8.3	2.85 ± 2.97	−0.16 ± 2.73	2.4 ± 3.5	0.19 ± 3.6	11.9 ± 10.7	0.13 ± 11.9	14.9 ± 10.8	−3.6 ± 9.6 ^2^
	Diet	17.1 ± 6.7	1.2 ± 6.6	2.5 ± 2.5	2.0 ± 3.9 ^2,b^	3.2 ± 3.9	1.9 ± 4.5 ^2,b^	10.4 ± 8.9	2.1 ± 8.7	13.2 ± 11.8	4.2 ± 10.2 ^2,b^
Fluid (mL/day)	Control	1756 ± 542	64.9 ± 552	2565 ± 623	−80.5 ± 633	2438 ± 614	−71.7 ± 607	2405 ± 605	−68.9 ± 603	2367 ± 553	25.3 ± 595
	Diet	1901 ± 588	144 ± 584 ^1^	2721 ± 732	66.7 ± 719 ^b^	2356 ± 561	116 ± 574 ^b^	2451 ± 593	127 ± 580 ^b^	2189 ± 529	378 ± 546 ^2,b^
Alcohol (g/day)	Control	8.1 ± 9.9	−1.1 ± 9.6	10.9 ± 10.8	−1.4 ± 10.7	13.4 ± 12.5	0.0 ± 12.8	3.9 ± 6.4	−0.7 ± 6.1	10.6 ± 11.7	−0.1 ± 11.7
	Diet	9.0 ± 9.9	−2.6 ± 8.8 ^1^	9.6 ± 10.1	−1.6 ± 9.4	11.9 ± 11.2	0.8 ± 11.5	3.7 ± 5.8	−1.7 ± 4.9 ^1^	9.5 ± 12.1	−3.1 ± 10.4 ^b^
Salt (mg/day)	Control	1731 ± 545	−3.2 ± 543	2376 ± 879	−162 ± 801	2435 ± 670	−175 ± 677 ^1^	3495 ± 1118	−68.5 ± 1110	3289 ± 2282	−489 ± 1711 ^1^
	Diet	1809 ± 698	−173 ± 615 ^1,a^	2357 ± 679	−295 ± 669 ^2^	2323 ± 618	−54.5 ± 577	3523 ± 1143	−122 ± 1144	2968 ± 2988	−695 ± 2166 ^1^
Sweets (g/day)	Control	43.6 ± 51.2	2.3 ± 56.7	137 ± 166	−11.7 ± 156	83.6 ± 93.5	−16.8 ± 82.4	96.3 ± 111	−10.6 ± 97.7	51.1 ± 77.9	0.4 ± 91.8
	Diet	55.5 ± 67.7	−16.6 ± 61.7 ^1,a^	132 ± 129	−49.8 ± 107 ^2,b^	79.5 ± 102	−5.3 ± 106	104 ± 113	−21.6 ± 102	56.4 ± 58.2	−23.2 ± 51.6 ^2,a^
Vitamin D, *n* (%)	Control	25 (21.9)	17 (14.9) ^1^	12 (9.5)	7 (5.6)	15 (12)	8 (6.6)	22 (20.8)	17 (16.0) ^2^	7 (7.0)	8 (8.0)
	Diet	22 (17.3)	83 (65.4) ^2,b^	11 (8.7)	108 (85.7) ^2,b^	16 (13.7)	88 (75.2) ^2,b^	22 (19.3)	83 (72.7) ^2,b^	13 (14.9)	68 (78.2) ^2,b^
NU-AGE index	Control	86.9 ± 14.9	2.5 ± 14.7	79.9 ± 15.2	6.7 ± 15.5 ^2^	72.9 ± 15.8	3.6 ± 16.6	84.7 ± 17.2	0.53 ± 16.5	91.2 ± 12.9	−5.4 ± 13.4 ^2^
	Diet	82.5 ± 15.4	21.2 ± 14.4 ^2,b^	81.5 ± 12.7	20.8 ± 13.9 ^2,b^	76.1 ± 15.4	20.3 ± 15.5 ^2,b^	86.1 ± 14.8	21.4 ± 15.2 ^2,b^	88.2 ± 15.8	35.6 ± 16.0 ^2,b^

^1,2^ Significant change from baseline to follow-up (^1^: *p* < 0.05, ^2^: *p* < 0.01); ^a,b^ Significant difference in change in the NU-AGE diet group compared to the control group (^a^: *p* < 0.05, ^b^: *p* < 0.01).
